# Supplementary material for: Recognition of ATT Triplex and DNA:RNA Hybrid Structures by Benzothiazole Ligands
Source: Biomolecules. 2022 Feb 27;12(3):374. doi: 10.3390/biom12030374 (PMC8945811; doi:10.3390/biom12030374)
Supplement: Supplementary file 1 [file biomolecules-12-00374-s001.zip › biomolecules-1600173-supplementary.pdf]

Supporting information

Recognition of ATT triplex and DNA:RNA hybrid structures by benzothiazole ligands

Iva Zonjić [1], Lidija Marija Tumir [1], Ivo Crnolatac [1], Filip Šupljika [3], Livio Racané [4], Sanja Tomić [2] and Marijana Radić Stojković [1,*]

[1] Laboratory for Biomolecular Interactions and Spectroscopy, Division of Organic Chemistry and Biochemistry, Ruđer Bošković Institute, HR 10002 Zagreb, P.O.B. 180, Croatia; Iva.Zonjic@irb.hr (I.Z.); tumir@irb.hr (L-M.T.); icrnolat@irb.hr (I.C.)
[2] Laboratory for Protein Biochemistry and Molecular Modelling, Division of Organic Chemistry and Biochemistry, Ruđer Bošković Institute, HR 10002 Zagreb, P.O.B. 180, Croatia; Sanja.Tomic@irb.hr (S.T.)
[3] Faculty of Food Technology and Biotechnology, University of Zagreb, Pierrotijeva 6, 10002 Zagreb, Croatia; fsupljika@pbf.hr (F.Š.)
[4] Department of Applied Chemistry, Faculty of Textile Technology, University of Zagreb, Prilaz baruna Filipovića 28a, 10000 Zagreb, Croatia; lracane@ttf.hr (L.R.)

Contents

1. Spectroscopic characterization of benzothiazoles 1-9 in aqueous solutions.

2. Interactions of 1-9 with ds-polynucleotides in neutral medium (pH=7.0)

2.1. Competition dialysis

2.2. Fluorimetric titrations

2.3. Isothermal titration calorimetry

2.4. Thermal melting experiments

2.5. Circular dichroism (CD) titrations

3. Characterization of 1-9 compounds (NMR, elemental analysis, ESI)

1. Spectroscopic characterization of benzothiazoles 1-9 in aqueous solutions.

1.1. Solubility

All studied benzothiazoles were dissolved in redistilled water (c=3-5 × 10^{-3} mol dm^{-3}) or aqueous buffer (sodium cacodylate/HCl buffer, I = 0.05 mol dm^{-3}).

1.2. UV/Vis spectra, stability

Buffered solutions of studied compounds were stable for days at 4-8°C (refrigerator). The absorbances of buffered solutions of studied benzothiazole compouds were propor-tional to their concentrations up to c =2 × 10^{-5} mol dm^{-3} (Figures S1–S10).

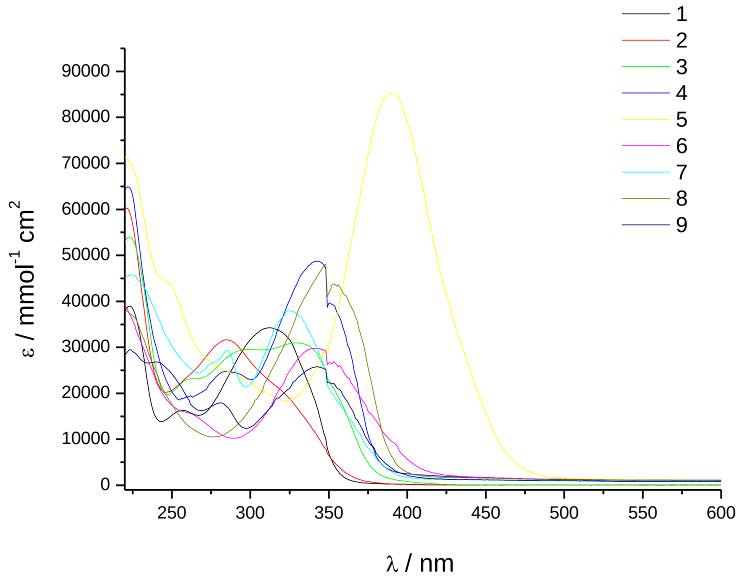

Figure S1. UV/Vis spectra of **1-9** at c = 1.6 × 10^{-5} - 2 × 10^{-5} mol dm^{-3}; pH=7, sodium cacodylate/HCl buffer, I = 0.05 mol dm^{-3}.

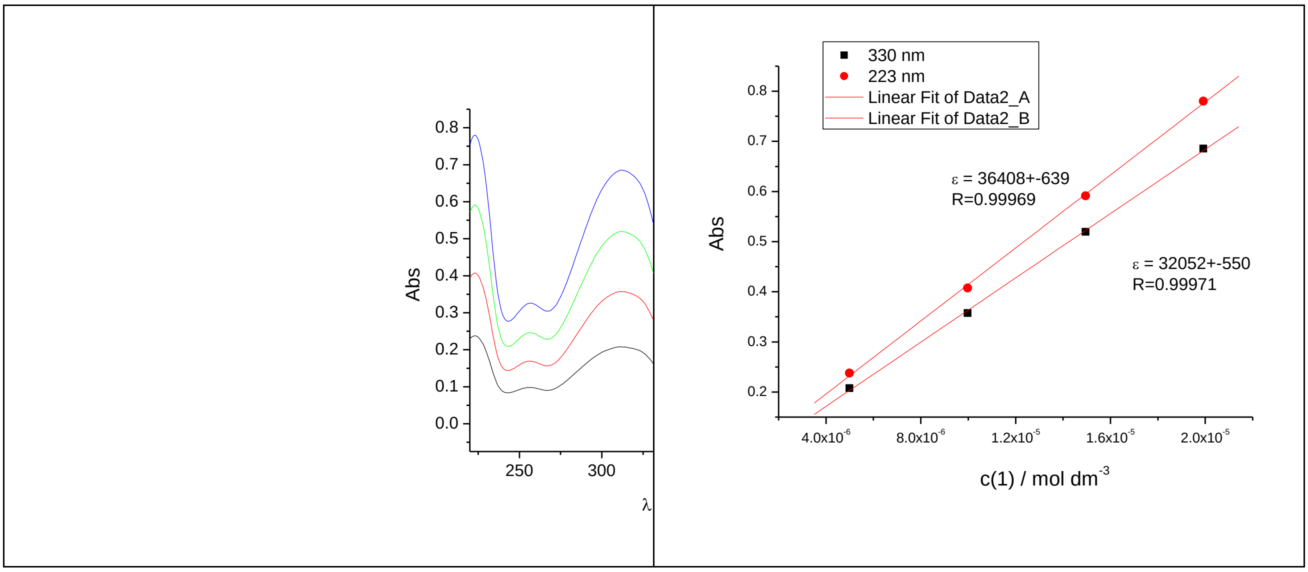

Figure S2. UV/Vis spectra changes of **1** at different concentrations (concentration range from 5 × 10^{-6}- 2 × 10^{-5} mol dm^{-3}) (left); linear dependence (—) of the absorbance at 223 and 330 nm (■) on the **1** concentration (right); (pH=7, sodium cacodylate buffer, *I*=0,05 M).

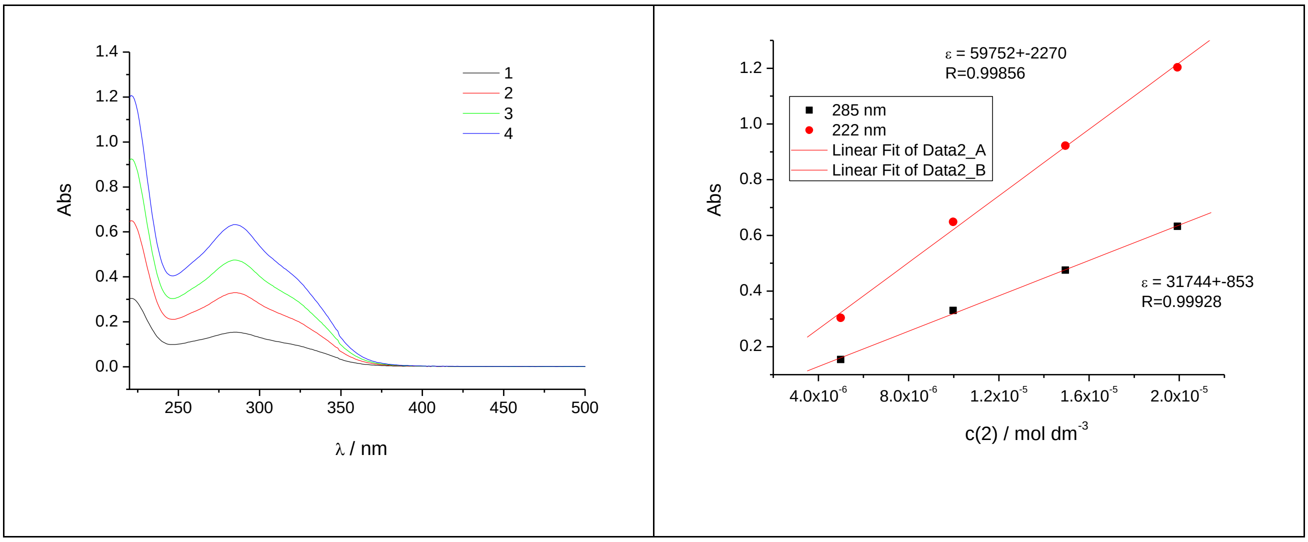

Figure S3. UV/Vis spectra changes of **2** at different concentrations (concentration range from 5 × 10^{-6}- 2 × 10^{-5} mol dm^{-3}) (left); linear dependence (—) of the absorbance at 222 and 385 nm (■) on the **2** concentration (right); (pH=7, sodium cacodylate buffer, *I*=0,05 M).

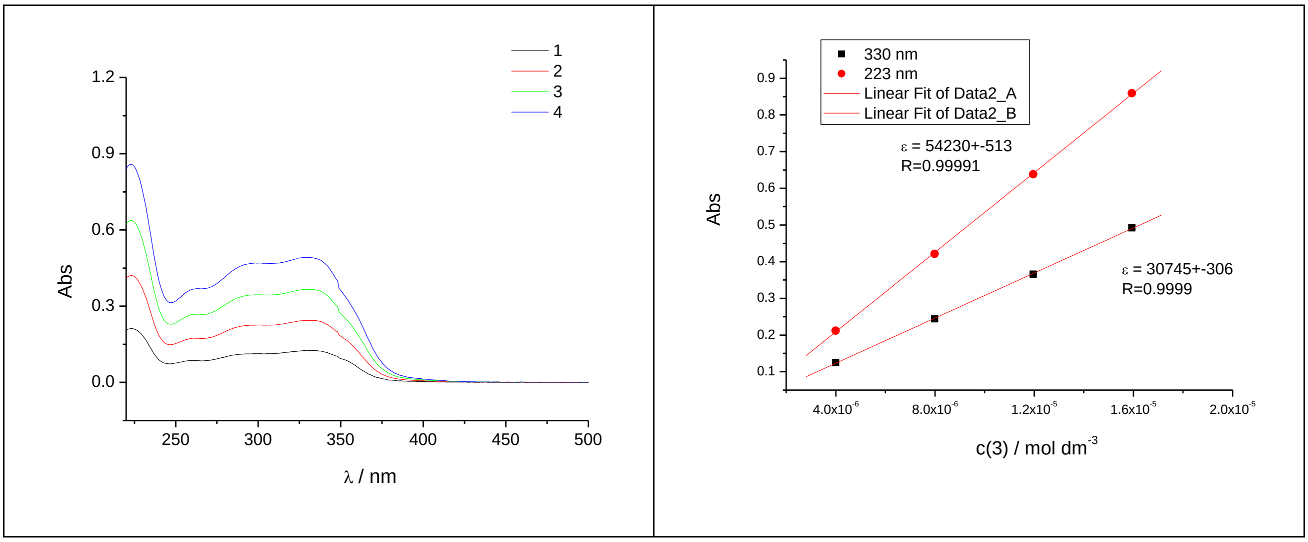

Figure S4. UV/Vis spectra changes of **3** at different concentrations (concentration range from 5 × 10^{-6}- 2 × 10^{-5} mol dm^{-3}) (left); linear dependence (—) of the absorbance at 223 and 330 nm (■) on the **3** concentration (right); (pH=7, sodium cacodylate buffer, *I*=0,05 M).

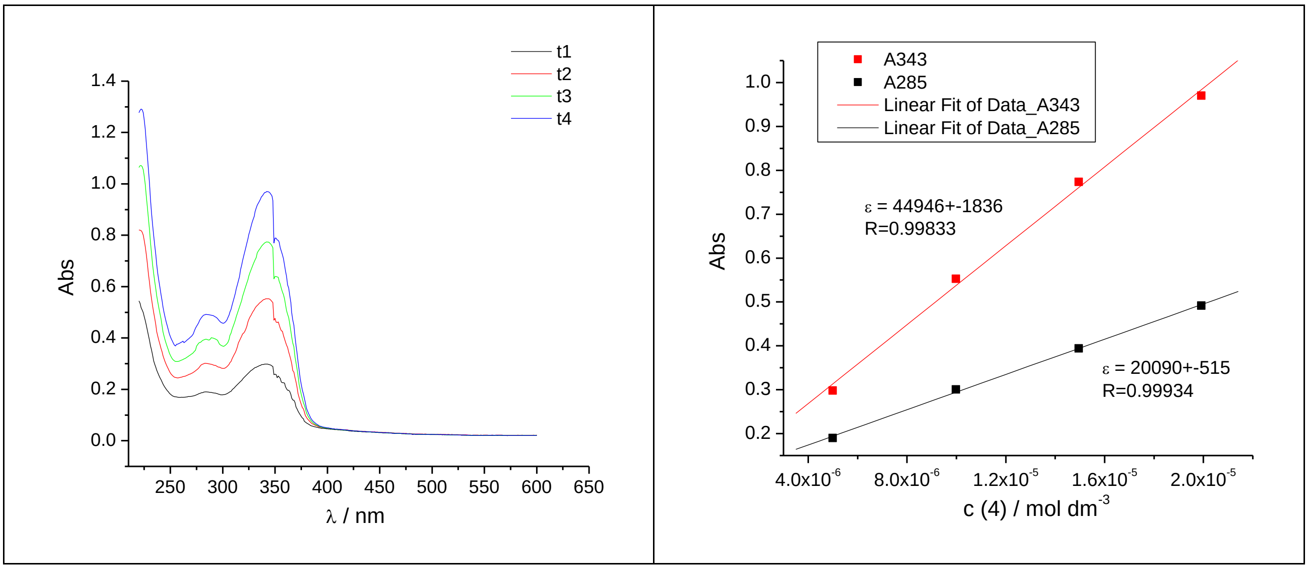

Figure S5. UV/Vis spectra changes of **4** at different concentrations (concentration range from 5×10^{-6}- 2×10^{-5} mol dm^{-3}) (left); linear dependence (—) of the absorbance at 285 and 343 nm (■) on the **4** concentration (right); (pH=7, sodium cacodylate buffer, *I*=0,05 M).

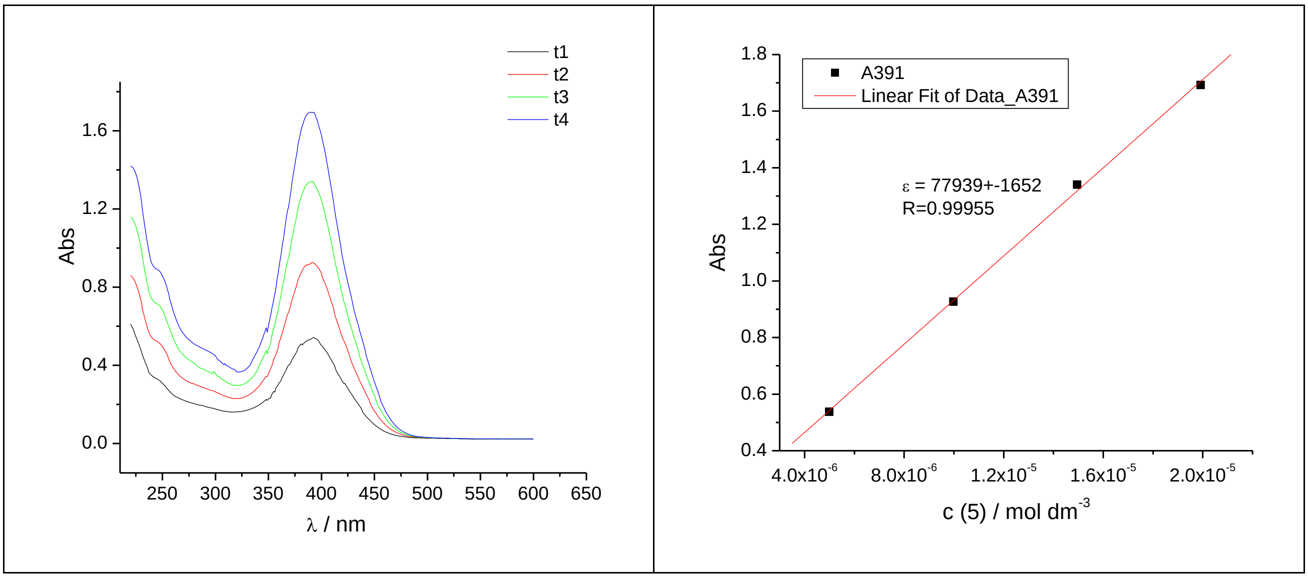

Figure S6. UV/Vis spectra changes of **5** at different concentrations (concentration range from 5×10^{-6}- 2×10^{-5} mol dm^{-3}) (left); linear dependence (—) of the absorbance at 391 nm (■) on the **5** concentration (right); (pH=7, sodium cacodylate buffer, *I*=0,05 M).

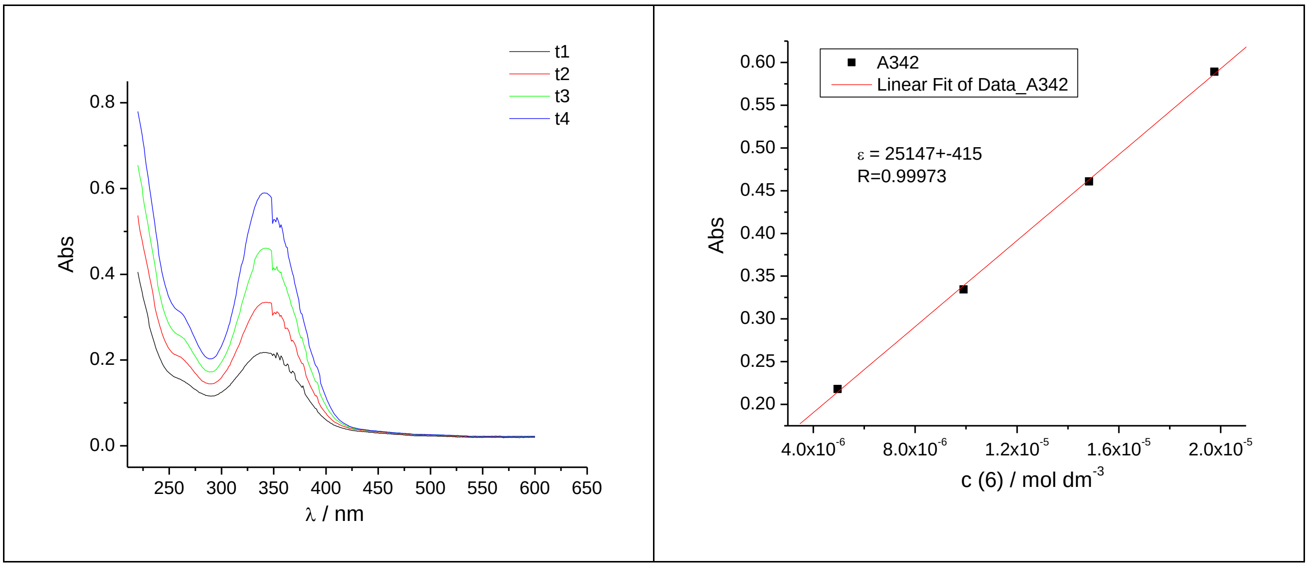

Figure S7. UV/Vis spectra changes of **6** at different concentrations (concentration range from 5 × 10^{-6} - 2 × 10^{-5} mol dm^{-3}) (left); linear dependence (—) of the absorbance at 342 nm (■) on the **6** concentration (right); (pH=7, sodium cacodylate buffer, *I*=0,05 M).

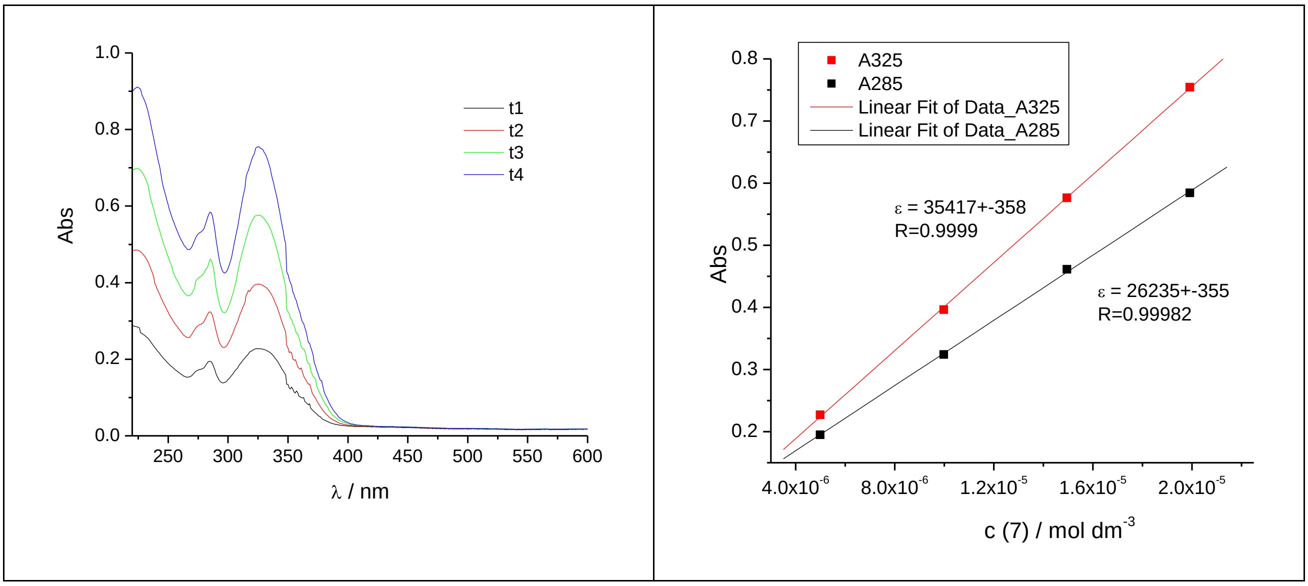

Figure S8. UV/Vis spectra changes of **7** at different concentrations (concentration range from 5 × 10^{-6} - 2 × 10^{-5} mol dm^{-3}) (left); linear dependence (—) of the absorbance at 285 and 325 nm (■) on the **7** concentration (right); (pH=7, sodium cacodylate buffer, *I*=0,05 M).

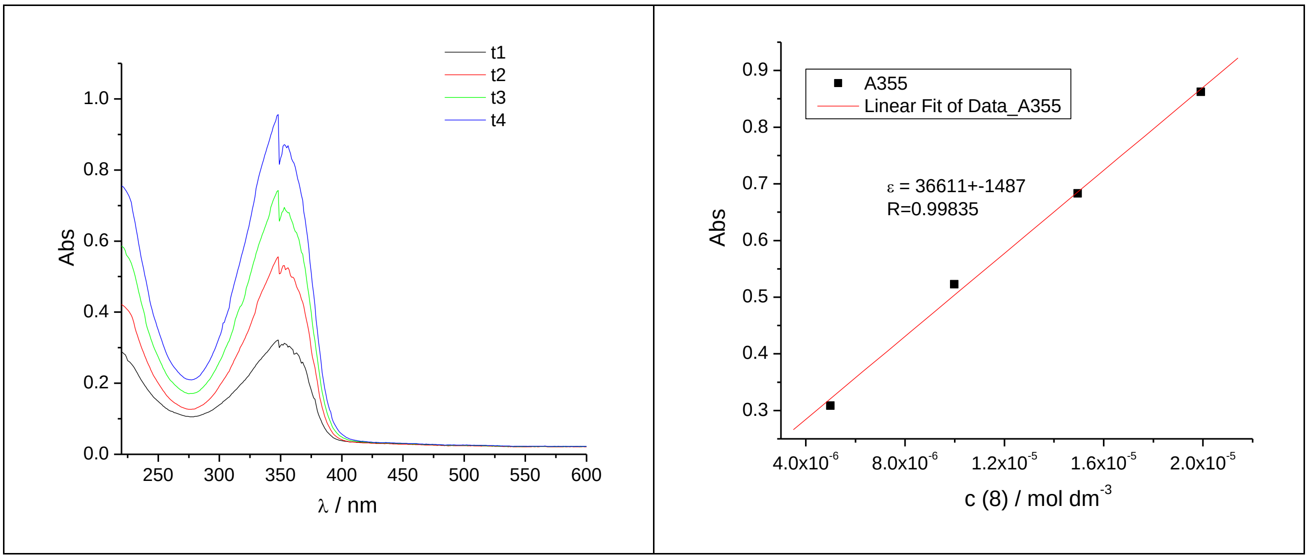

Figure S9. UV/Vis spectra changes of **8** at different concentrations (concentration range from 5 × 10^{-6}- 2 × 10^{-5} mol dm^{-3}) (left); linear dependence (—) of the absorbance at 355 nm (■) on the **8** concentration (right); (pH=7, sodium cacodylate buffer, *I*=0,05 M).

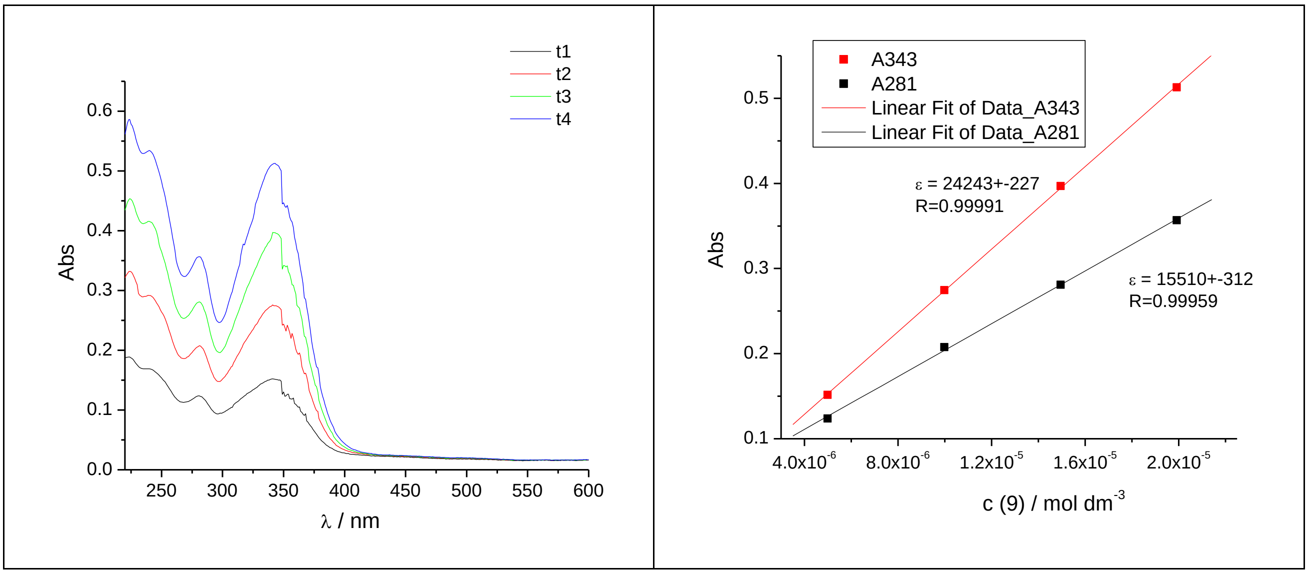

Figure S10. UV/Vis spectra changes of **9** at different concentrations (concentration range from 5 × 10^{-6}- 2 × 10^{-5} mol dm^{-3}) (left); linear dependence (—) of the absorbance at 281 and 343 nm (■) on the **9** concentration (right); (pH=7, sodium cacodylate buffer, *I*=0,05 M).

1.3. Fluorescence spectra

The emission intensities of buffered aqueous solutions (sodium cacodylate buffer, I = 0.05 mol dm^{-3}, pH = 7) of studied compounds are proportional to their concentrations up to $c = 2 \times 10^{-6}$ mol dm^{-3} (Figures S11-S20).

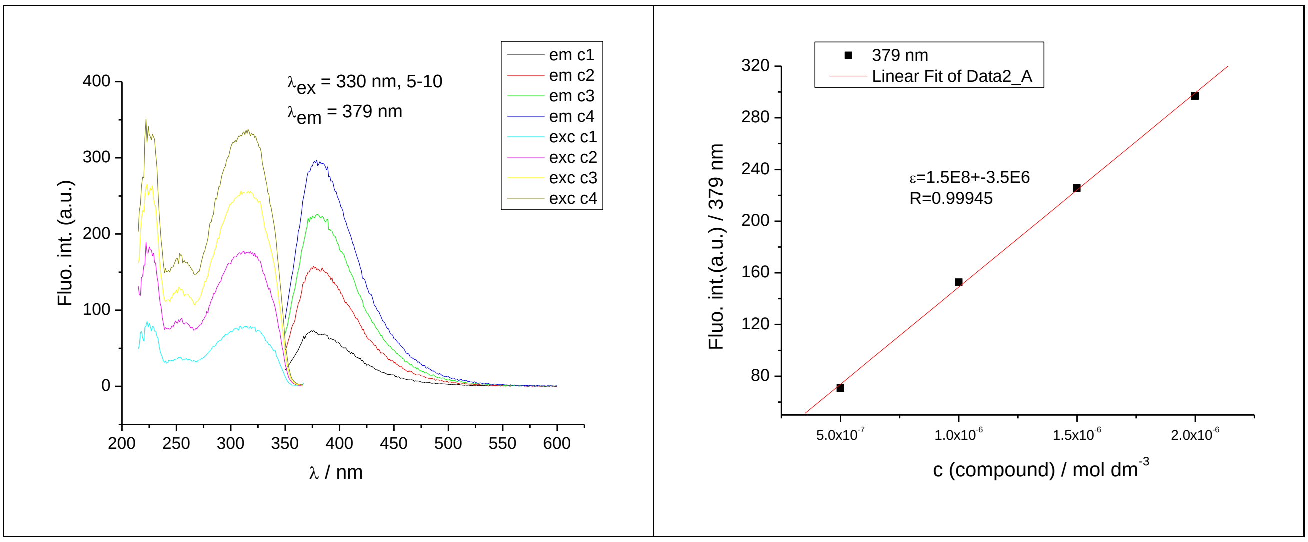

Figure S11. Emission and excitation spectra changes of **1** at different concentrations at λ_{exc}=330 nm (concentration range from 5×10^{-7}- 2×10^{-6} mol dm^{-3}) at pH=7.0, sodium cacodylate buffer, I=0.05 mol dm^{-3}.

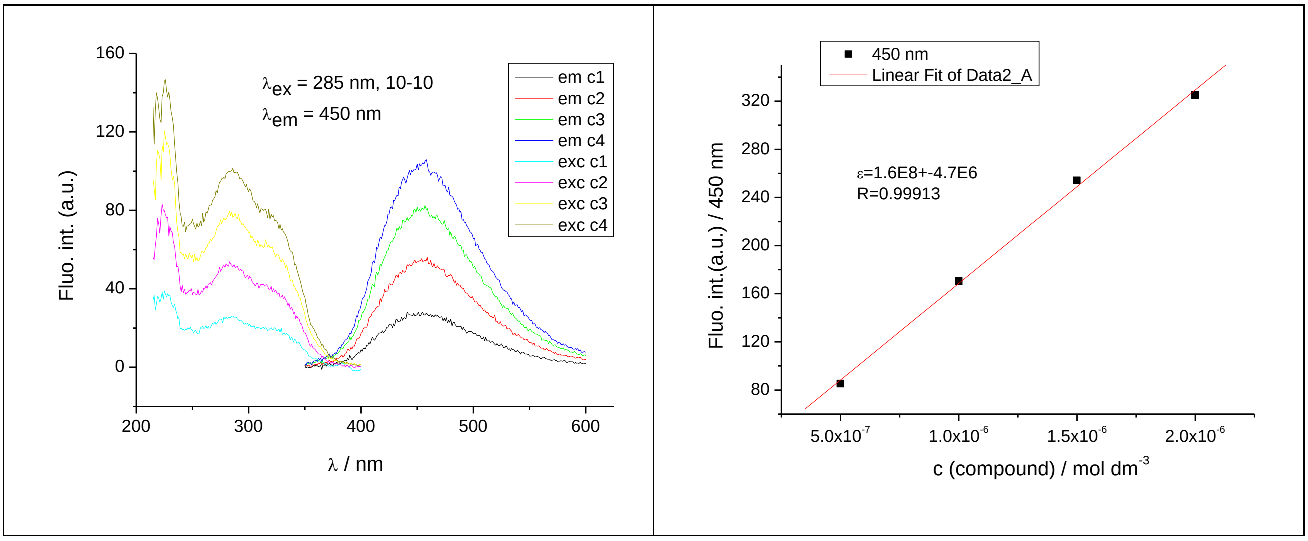

Figure S12. Emission and excitation spectra changes of **2** at different concentrations at λ_{exc}=285 nm (concentration range from 5×10^{-7}- 2×10^{-6} mol dm^{-3}) at pH=7.0, sodium cacodylate buffer, I=0.05 mol dm^{-3}.

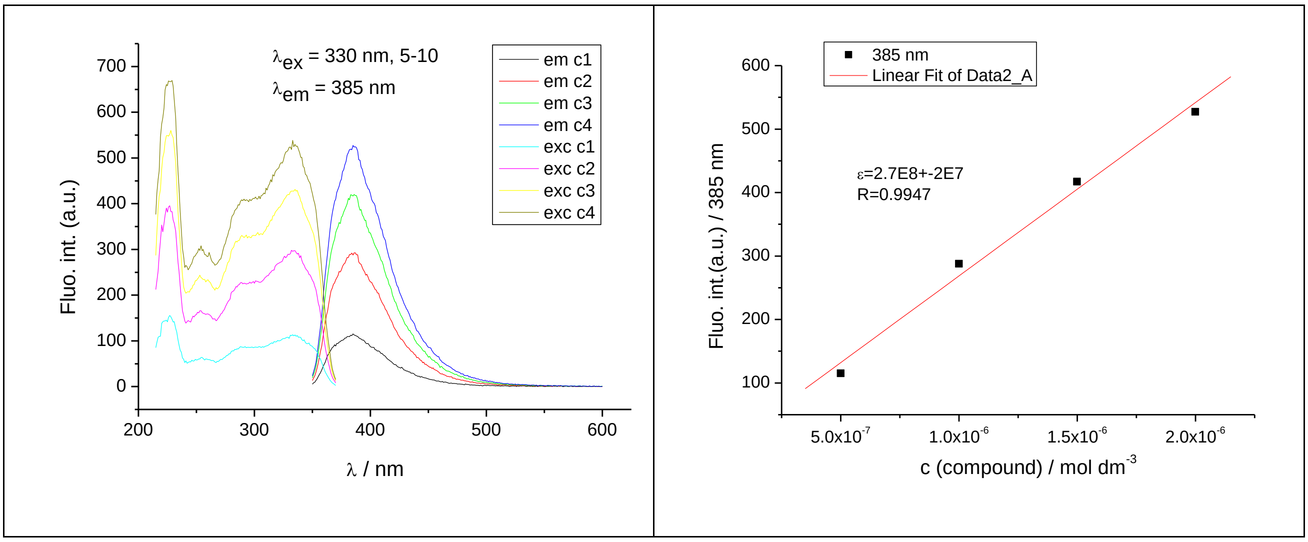

Figure S13. Emission and excitation spectra changes of **3** at different concentrations at λ_{exc}=330 nm (concentration range from 5 × 10^{-7}- 2 × 10^{-6} mol dm^{-3}) at pH=7.0, sodium cacodylate buffer, *I*=0.05 mol dm^{-3}.

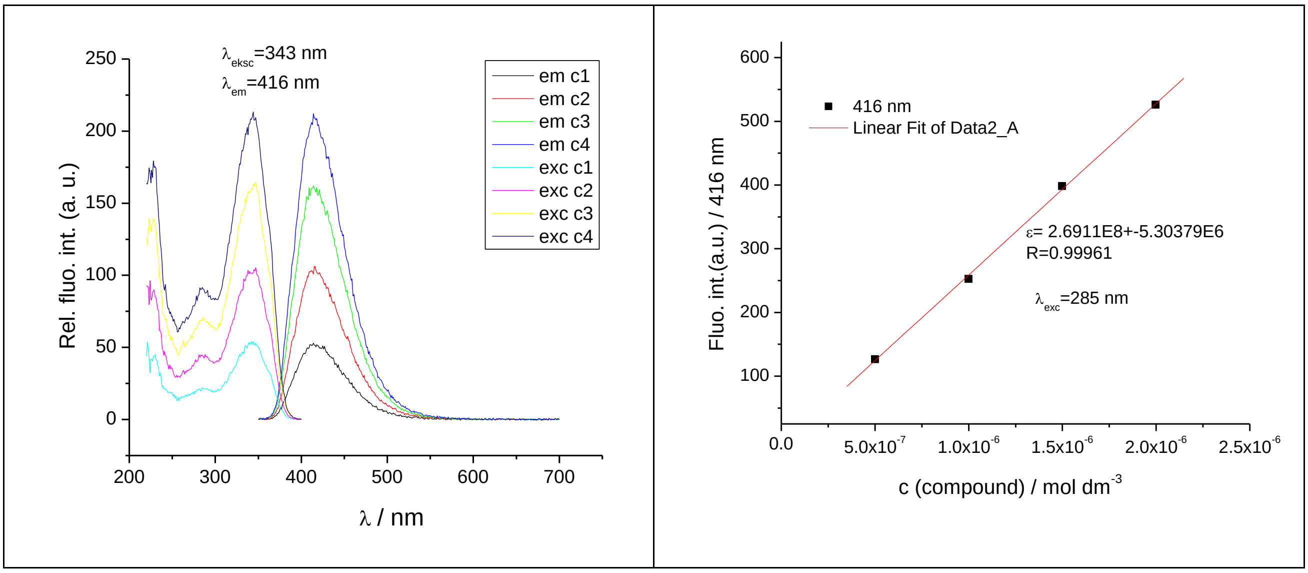

Figure S14. Emission and excitation spectra changes of **4** at different concentrations at λ_{exc}=343 nm (concentration range from 5 × 10^{-7}- 2 × 10^{-6} mol dm^{-3}) at pH=7.0, sodium cacodylate buffer, *I*=0.05 mol dm^{-3}.

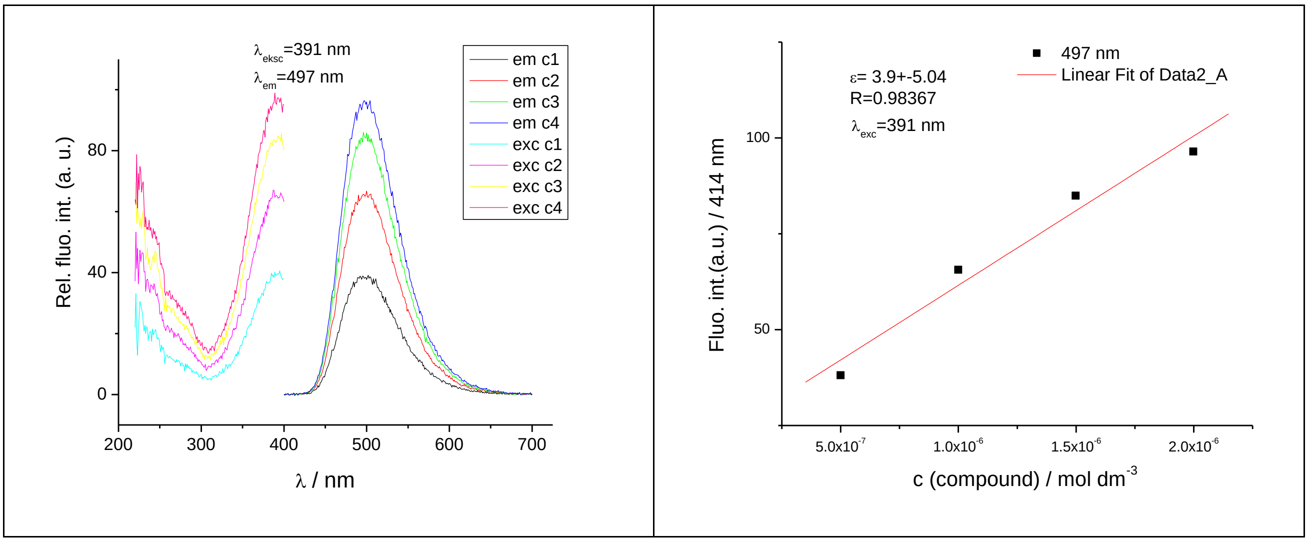

Figure S15. Emission and excitation spectra changes of **5** at different concentrations at λ_{exc}=391 nm (concentration range from 5 × 10^{-7}- 2 × 10^{-6} mol dm^{-3}) at pH=7.0, sodium cacodylate buffer, *I*=0.05 mol dm^{-3}.

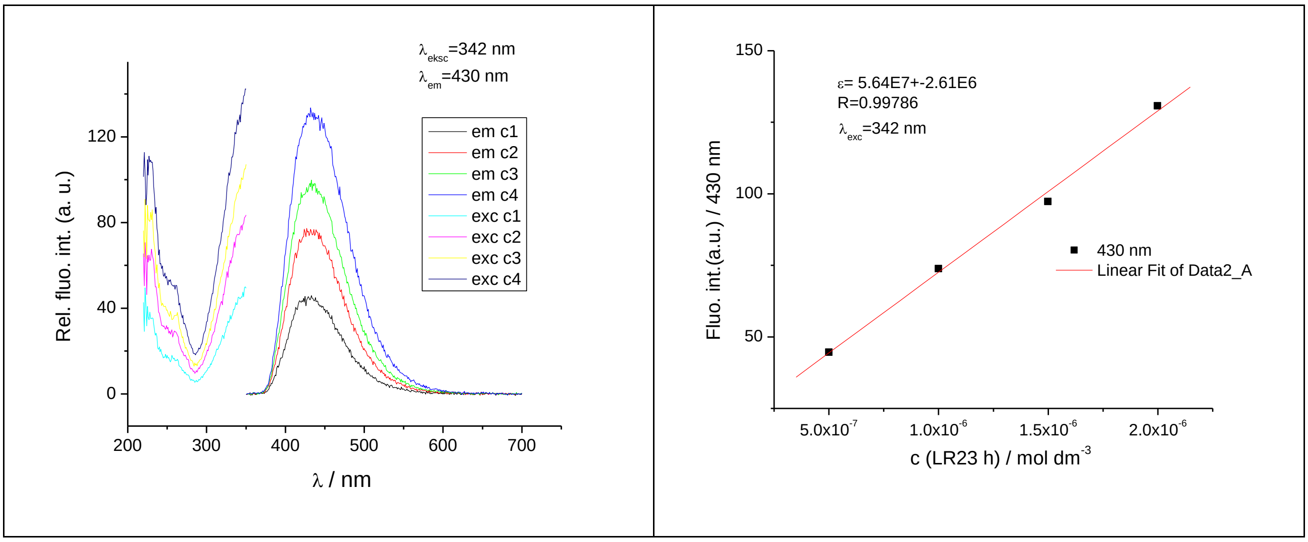

Figure S16. Emission and excitation spectra changes of **6** at different concentrations at λ_{exc}=342 nm (concentration range from 5 × 10^{-7}- 2 × 10^{-6} mol dm^{-3}) at pH=7.0, sodium cacodylate buffer, *I*=0.05 mol dm^{-3}.

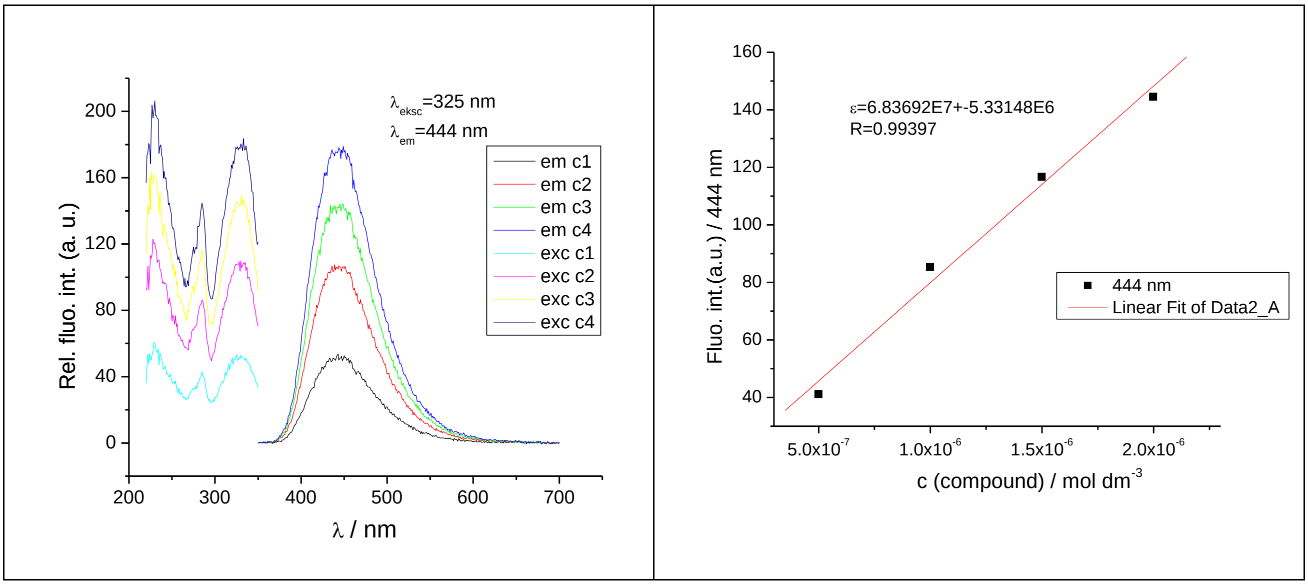

Figure S17. Emission and excitation spectra changes of **7** at different concentrations at λ_{exc}=325 nm (concentration range from 5 × 10^{-7}- 2 × 10^{-6} mol dm^{-3}) at pH=7.0, sodium cacodylate buffer, *I*=0.05 mol dm^{-3}.

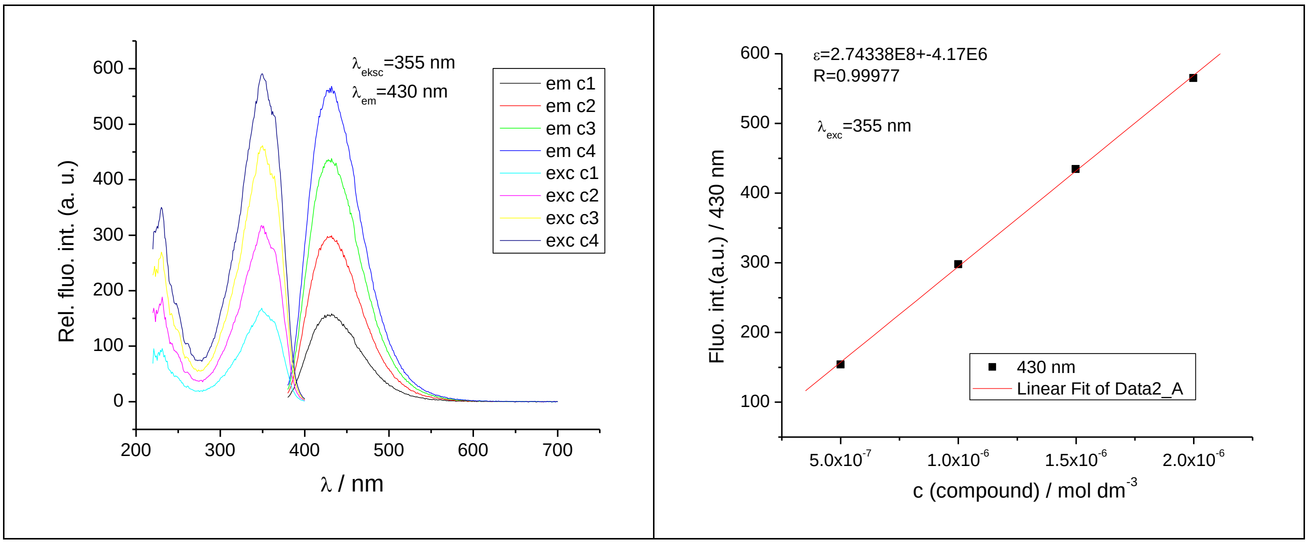

Figure S18. Emission and excitation spectra changes of **8** at different concentrations at λ_{exc}=355 nm (concentration range from 5 × 10^{-7}- 2 × 10^{-6} mol dm^{-3}) at pH=7.0, sodium cacodylate buffer, *I*=0.05 mol dm^{-3}.

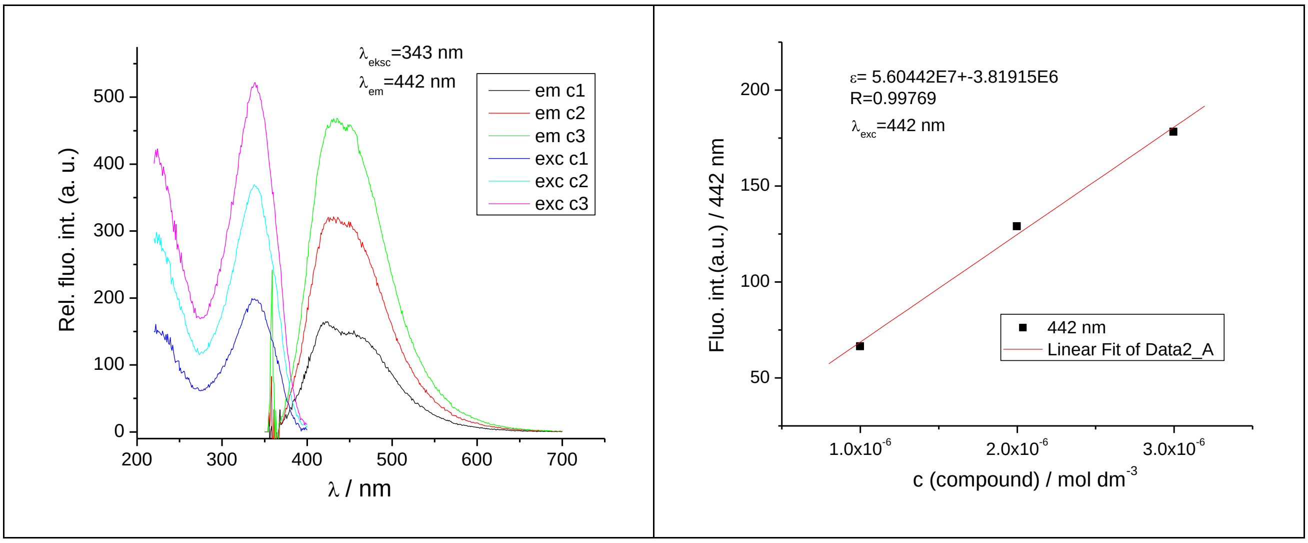

Figure S19. Emission and excitation spectra changes of **9** at different concentrations at λ_{exc}=343 nm (concentration range from 5 × 10^{-7}- 2 × 10^{-6} mol dm^{-3}) at pH=7.0, sodium cacodylate buffer, *I*=0.05 mol dm^{-3}.

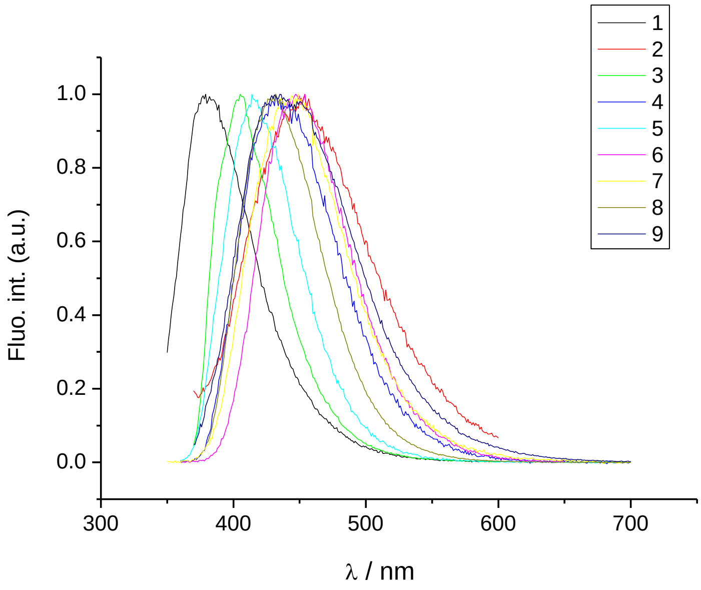

Figure S20. Normalized emission spectra of benzothiazoles **1-9**, *c*= 2 × 10^{-6} mol dm^{-3} at pH=7, Na cacodylate buffer, *I*=0.05 mol dm^{-3}.

2. Interactions of benzothiazoles with polynucleotides in neutral medium (pH=7.0)

2.1. Competition dialysis

Specificity sum, SS, is a metric that identifies compounds with high binding selectivity.

$$SS = \sum_i \frac{c_{b,i}}{c_{max}}$$

where $c_{b,i}$ is the amount of ligand bound and c_{max} is the maximum amount bound to any species.

The index i ranges from 1 to 13 in the current version of the assay, corresponding to the 13 different nucleic acid structures used. The SS value 1 pointed absolute selectivity for compound that binds only one polynucleotide, while SS value 13 pointed equal binding to all polynucleotides without any selectivity.

Metric c_{max} /SS includes information both on selectivity and binding affinity.

2.2. Fluorimetric titrations

Stability constants were determined by nonlinear Scatchard equation:

$$I = I_0 + ((I_{lim} - I_0) / (2\times c)) \times (c + n \times c_s + 1 / K_s - ((c + n \times c_s + 1 / K_s)^2 - 4 \times c \times n \times c_s)^{1/2})$$

where I is fluorescence intensity, while I_0 and I_{lim} denote fluorescence intensities of free and fully complexed ligand; c is concentration of free ligand; c_s is concentration of polynucleotide; n is ratio[bound ligand]/[polynucleotide] ; K_s is complex stability constant.

In Scatchard equation values of stability constant (Ks) and ratio (n=[bound compound] / [polynucleotide]) are highly mutually dependent and similar quality of fitting calculated to experimental data is obtained for ±20% variation for Ks and n; this variation can be considered as an estimation of the errors for the given binding constants. Given estimation was added to Footnote of Table 2.

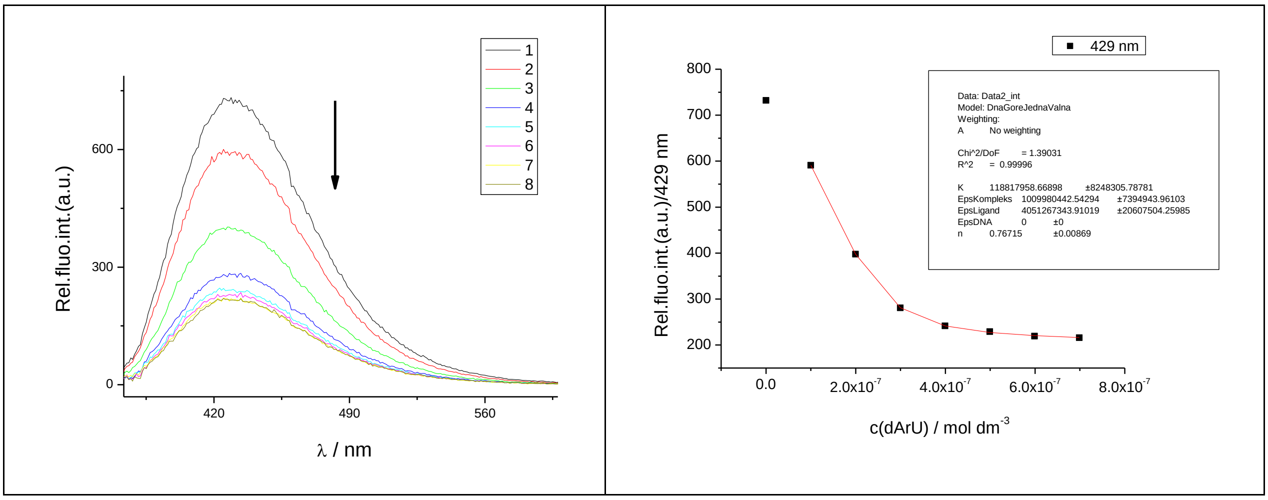

Figure S21. Left: Changes in fluorescence spectrum of **6** (c= 2 × 10^{-7} mol dm^{-3}, λ_{exc}=342 nm) upon titration with poly dA–poly rU (c= 9.9 × 10^{-8} – 7 × 10^{-7} mol dm^{-3}); Right: Experimental (●) and calculated (–) (by Scatchard eq.) fluorescence intensities of **6** at λ_{em} = 429 nm upon addition of poly dA–poly rU (pH = 7.0, Na cacodylate buffer, I = 0.2 mol dm^{-3} + 1mM EDTA).

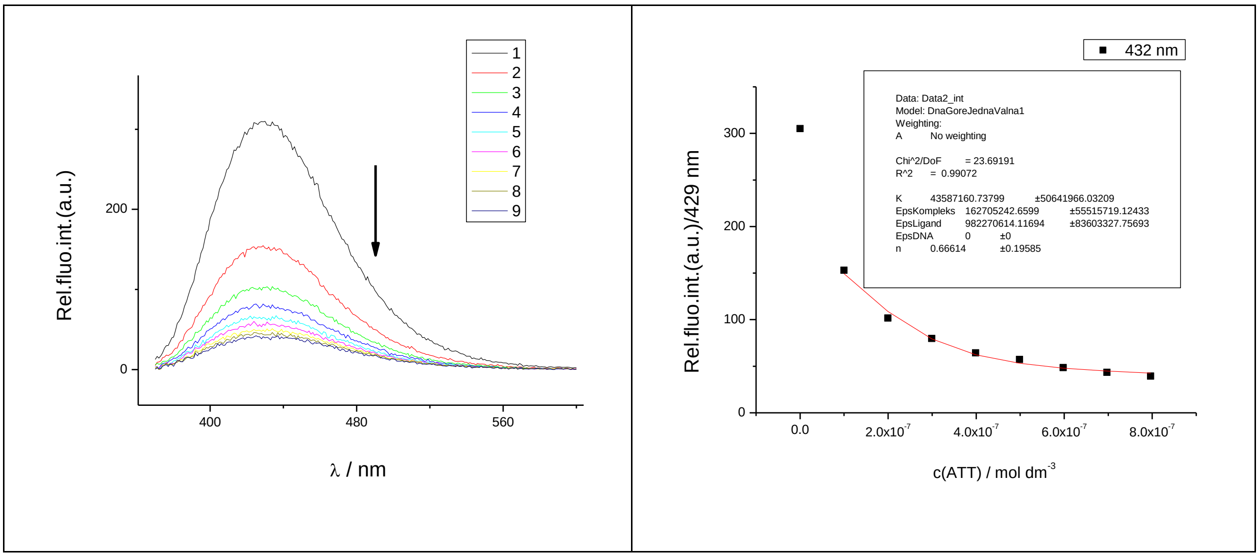

Figure S22. Left: Changes in fluorescence spectrum of **6** (c= 2 × 10^{-7} mol dm^{-3}, λ_{exc}=342 nm) upon titration with ATT triplex (c= 9.9 × 10^{-8} – 9.9 × 10^{-7} mol dm^{-3}); Right: Experimental (●) and calculated (–) (by Scatchard eq.) fluorescence intensities of **6** at λ_{em} = 432 nm upon addition of ATT triplex (pH = 7.0, Na cacodylate buffer, I = 0.05 mol dm^{-3} + 1mM EDTA).

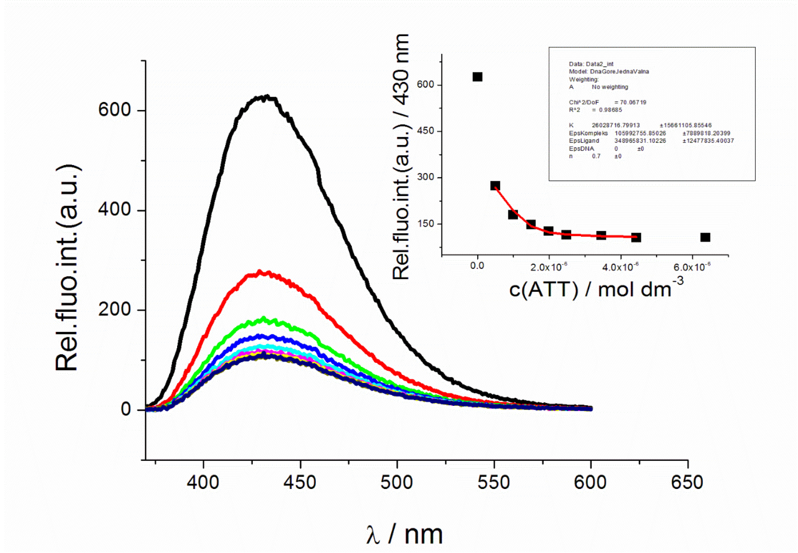

Figure S23. Changes in fluorescence spectrum of **6** (c= 1 × 10^{-6} mol dm^{-3}, λ_{exc}=342 nm) upon titration with 26mer ATT triplex (c= 5 × 10^{-7} – 6.36 × 10^{-6} mol dm^{-3}); Insert: Experimental (●) and calculated (–) (by Scatchard eq.) fluorescence intensities of **6** at λ_{em} = 430 nm upon addition of 26mer ATT triplex (pH = 7.0, Na cacodylate buffer, I = 0.05 mol dm^{-3} + 1mM EDTA).

2.3. Isothermal titration calorimetry

Titrations of ATT triplex and poly rA–poly dT hybrid with the compound **1** were performed using an isothermal titration microcalorimeter Microcal VP-ITC. One aliquot of 2 µL, twelve aliquots of 10 µL and eight aliquots of 20 µL of the compound 1 (c = 1.0 × 10^{-4} mol dm-3) were injected from rotating syringe (220 rpm) into the isothermal cell, equilibrated at 25.0 °C, containing 1.4406 mL of ATT or poly rA–poly dT (c = 3.0 × 10^{-5} mol dm^{-3}).

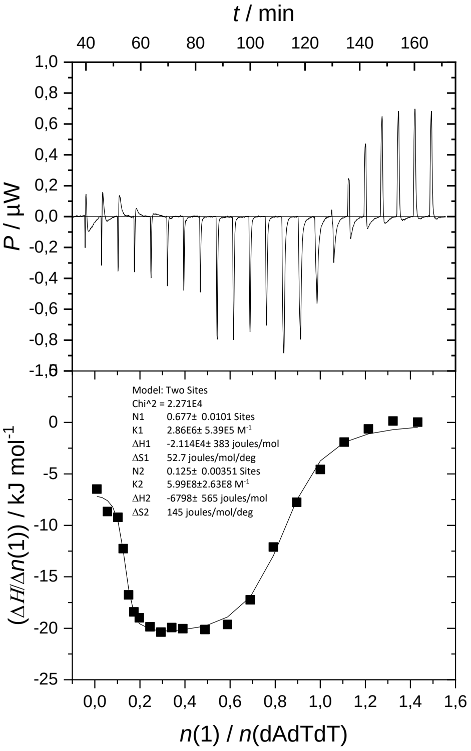

Figure S24. ITC experiment of ATT triplex titrated with **1**; experimental data (■) and calculated fit for model two sets of sites (–). Inset: raw titration data from the single injection of **1** into a solution of ATT triplex; [ATT] = 3.0 × 10^{-5} M; pH=7.0, Na-cacodylate buffer, I =0.05 mol dm^{-3} + 1mM EDTA.

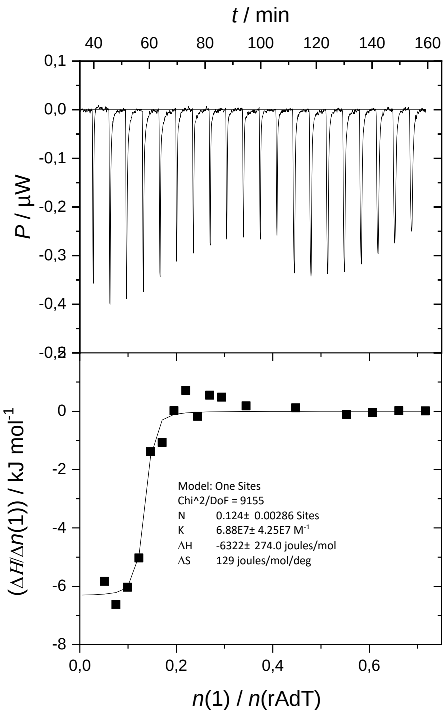

Figure S25. ITC experiment of poly rA–poly dT titrated with **1**; experimental data (■) and calculated fit for model two sets of sites (–). Inset: raw titration data from the single injection of **1** into a solution of poly rA–poly dT hybrid; [poly rA–poly dT] = 3.0 × 10^{-5} M; pH=7.0, Na-cacodylate buffer, *I* =0.05 mol dm^{-3} + 1mM EDTA.

2.4. Thermal denaturation experiments

$\Delta Tm = Tm$ (complex polynucleotide-dye) - Tm (polynucleotide)

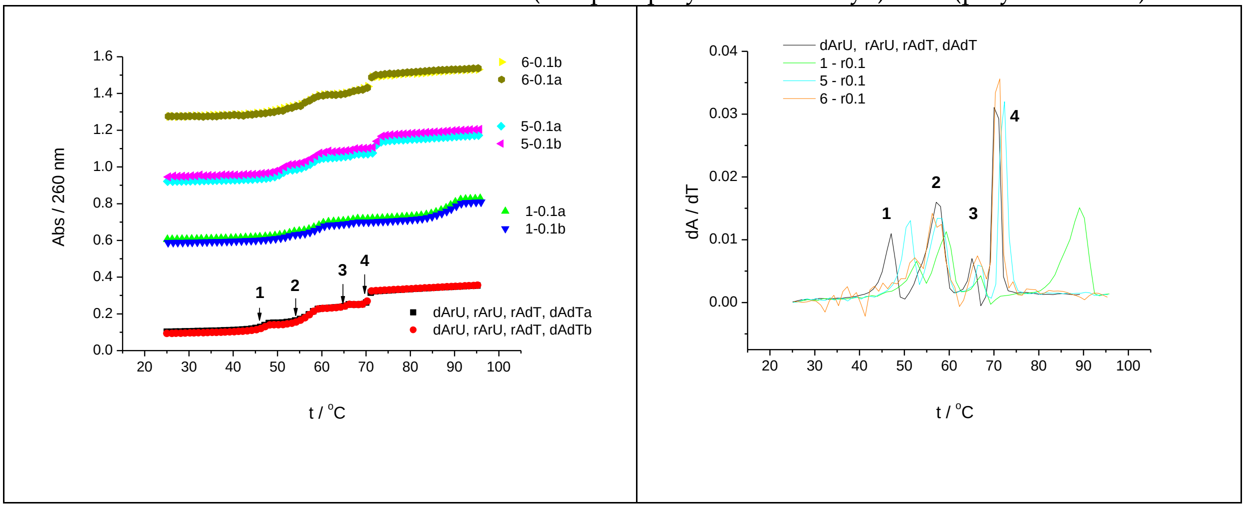

Figure S26. Left: Melting curves of polynucleotide mixtures (50mM sodium cacodylate buffer, 50mM NaCl, 1 mM EDTA): a DNA:RNA hybrid [poly dA–poly rU; peak 1], RNA [poly rA–poly rU; peak 2], an RNA:DNA hybrid [poly rA–poly dT; peak 3] and DNA [poly dA-poly dT; peak 4]. The concentration of each polynucleotide structure was 20 µM(bp); total polynucleotide concentration is 80 µM(bp). Effect of addition of ligand **1, 5** and **6** (2 µM) at ratio, ***r*** ([compound/ [polynucleotide])=0.025 to polynucleotide mixture was shown. Right: First derivative of absorbance at 260 nm in dependence of temperature.

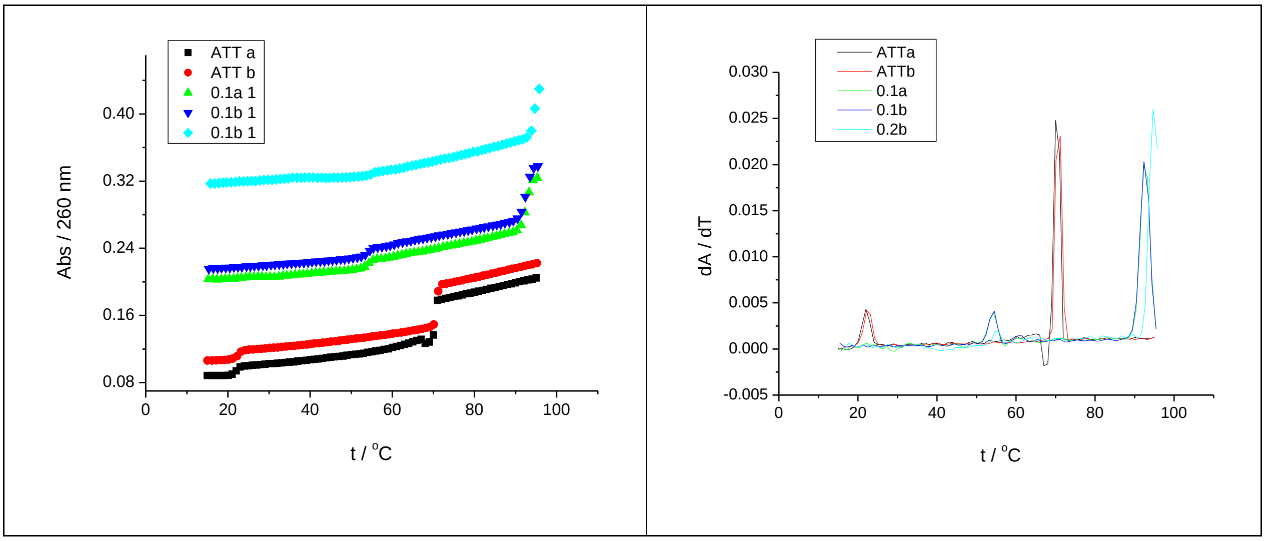

Figure S27. Left: Melting curve of ATT triplex upon addition of ratio, ***r*** ([compound/ [polynucleotide])=0.1 of **1** at pH = 7.0 (sodium cacodylate buffer with NaCl, I = 0.1 mol dm^{-3} + 1 mM EDTA);Right: First derivative of absorbance at 260 nm in dependence of temperature.

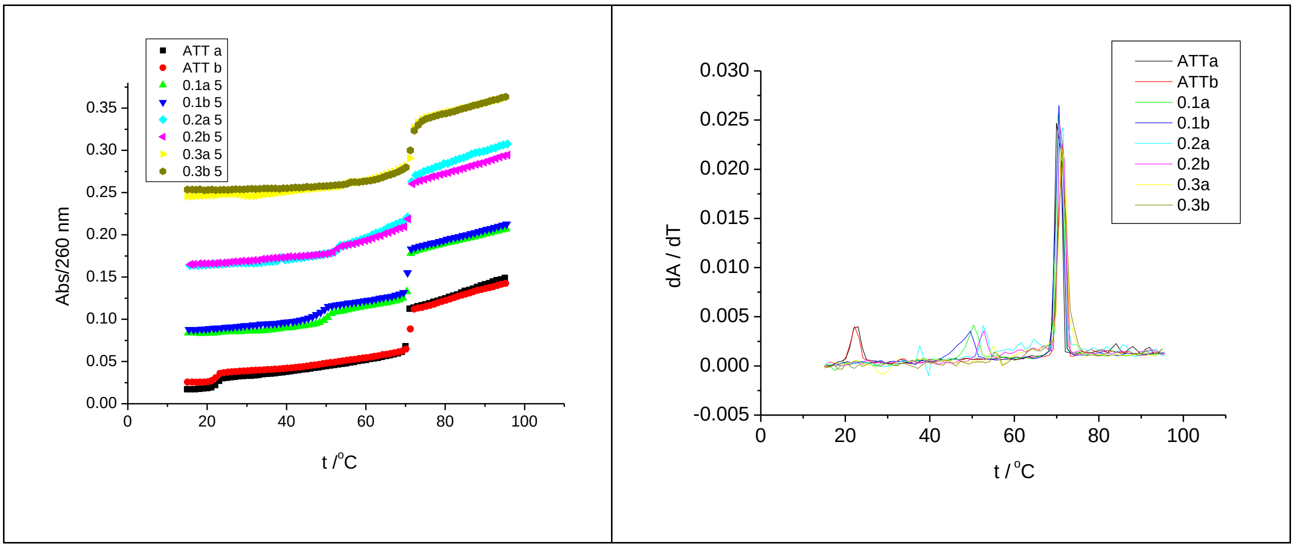

Figure S28. Left: Melting curve of ATT triplex upon addition of ratio, ***r*** ([compound/ [polynucleotide])=0.1 of **5** at pH = 7.0 (sodium cacodylate buffer with NaCl, I = 0.1 mol dm^{-3} + 1 mM EDTA);Right: First derivative of absorbance at 260 nm in dependence of temperature.

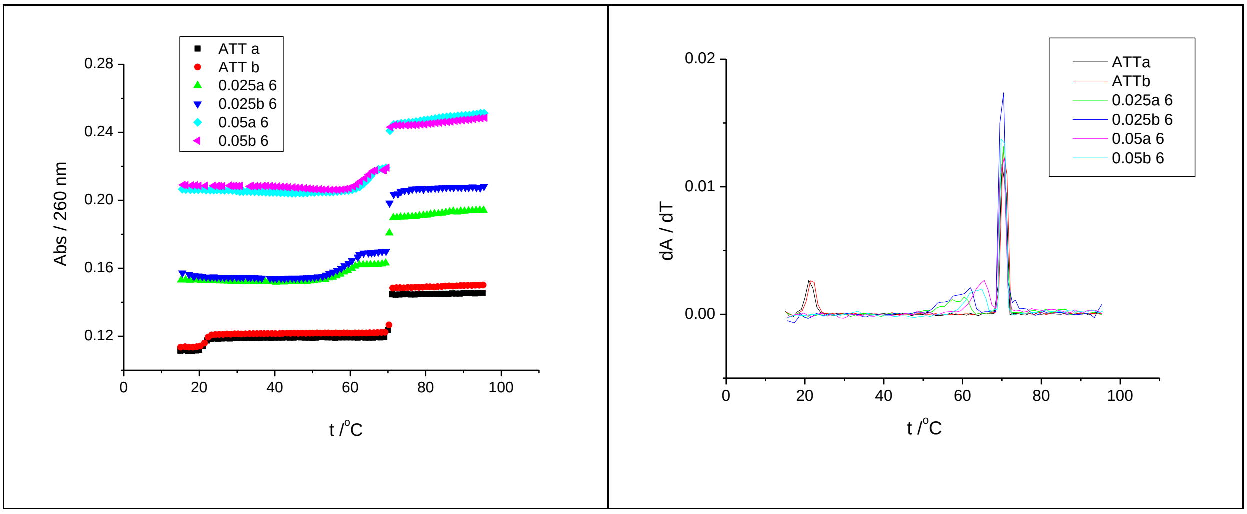

Figure S29. Left: Melting curve of ATT triplex upon addition of ratio, ***r*** ([compound/ [polynucleotide])=0.1 of **6** at pH = 7.0 (sodium cacodylate buffer with NaCl, I = 0.1 mol dm^{-3} + 1 mM EDTA);Right: First derivative of absorbance at 260 nm in dependence of temperature.

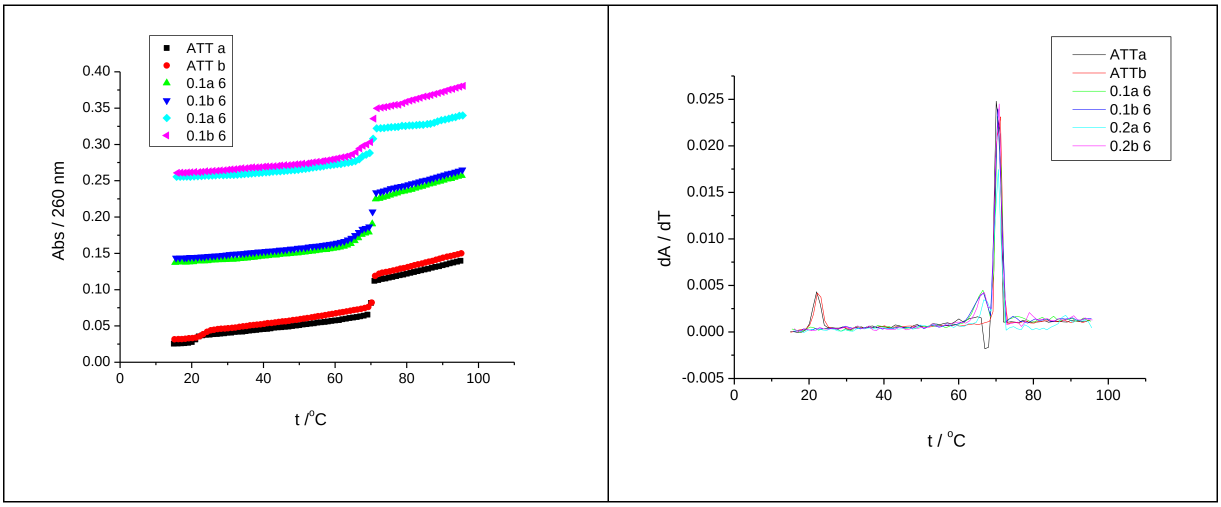

Figure S30. Left: Melting curve of ATT triplex upon addition of ratio, ***r*** ([compound/ [polynucleotide])=0.1 of **6** at pH = 7.0 (sodium cacodylate buffer with NaCl, I = 0.1 mol dm^{-3} + 1 mM EDTA);Right: First derivative of absorbance at 260 nm in dependence of temperature.

2.5. Circular dichroism (CD) experiments

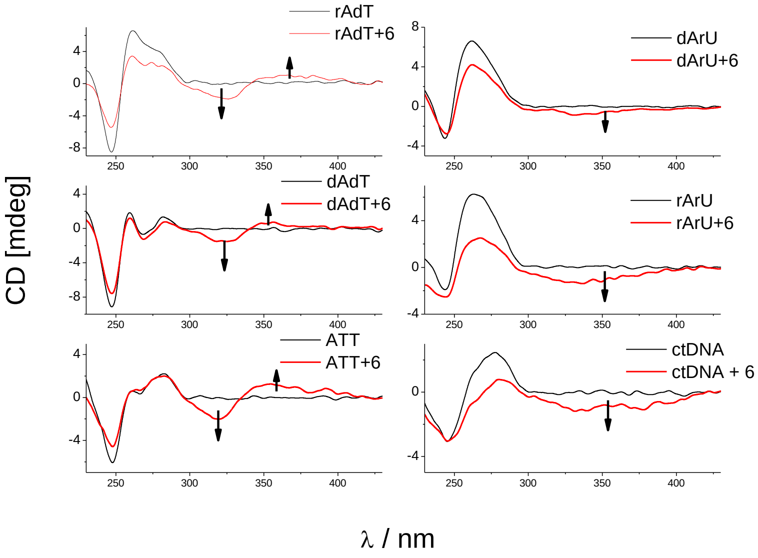

Figure S31. CD titrations of ATT triplex, poly rA–poly dT, poly rA–poly rU and poly dA–poly rU (c = 3.0 × 10^{-5} mol dm^{-3}) with **6** at molar ratios ***r*** = [compound] / [polynucleotide]=0.3 (pH = 7.0, buffer sodium cacodylate, I = 0.05 mol dm^{-3}+ 1 mM EDTA for all titrations except poly dA–poly rU (I = 0.2 mol dm^{-3} + 1 mM EDTA).

	ctDNA	poly rA – poly rU
**1**	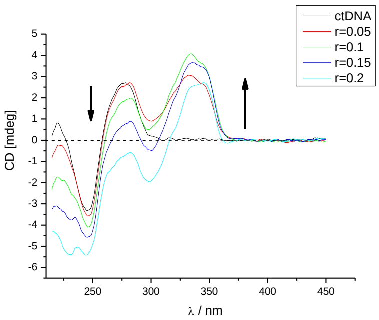	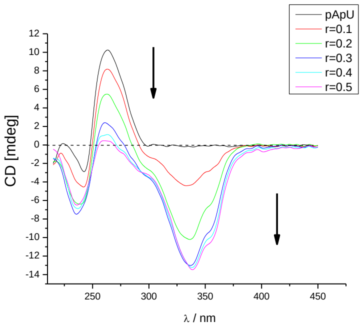
**3**	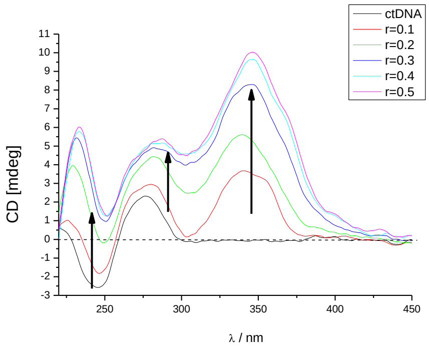	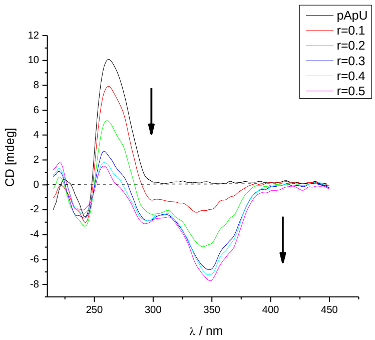
**5**	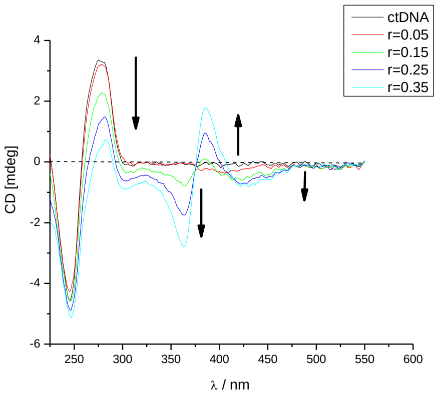	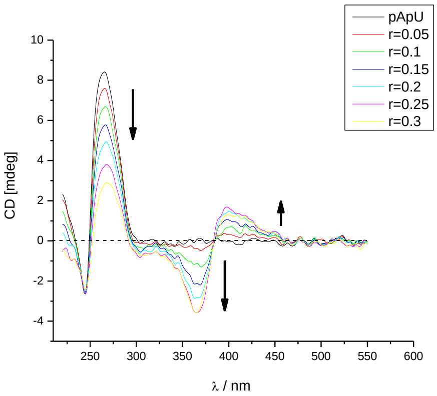
**6**	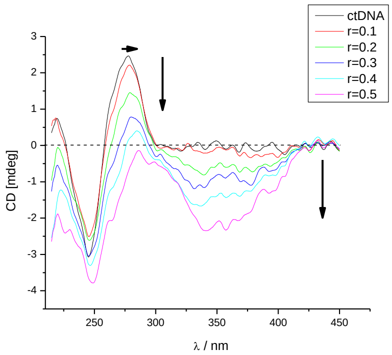	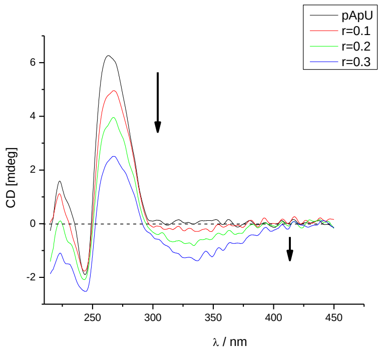

Figure S32. CD titrations of ctDNA ($c = 3.0 \times 10^{-5}$ mol dm^{-3}) and poly rA-poly rU ($c = 3.0 \times 10^{-5}$ mol dm^{-3}) with **1**, **3**, **5** and **6** at molar ratios ***r*** = [compound] / [polynucleotide] (pH = 7.0, buffer sodium cacodylate, $I = 0.05$ mol dm^{-3}).

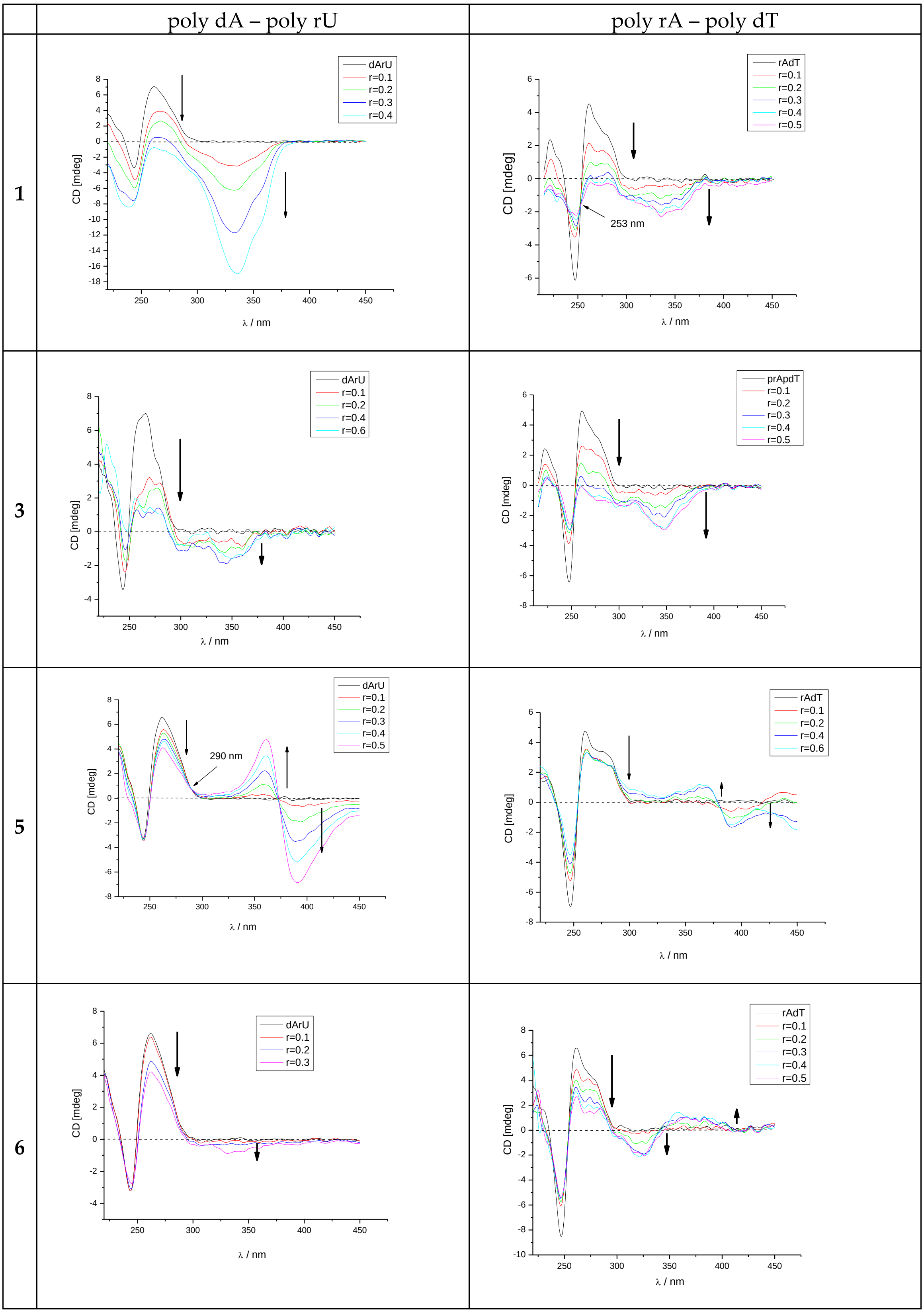

Figure S33. CD titrations of poly dA – poly rU ($c = 3.0 \times 10^{-5}$ mol dm^{-3}) and poly rA – poly dT ($c = 3.0 \times 10^{-5}$ mol dm^{-3}) with **1**, **3**, **5** and **6** at molar ratios ***r*** = [compound] / [polynucleotide] (pH = 7.0, buffer sodium cacodylate, $I = 0.05$ mol dm^{-3}).

<table>
<tr><th></th><th>poly dA – poly dT</th><th>ATT triplex</th></tr>
<tr><td>1</td><td>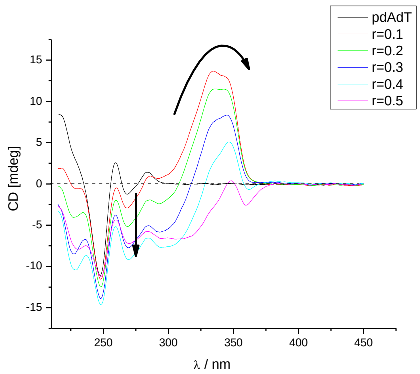
</td><td>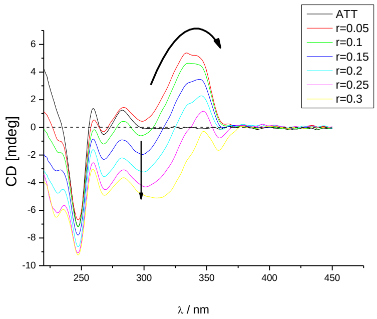
</td></tr>
<tr><td>3</td><td>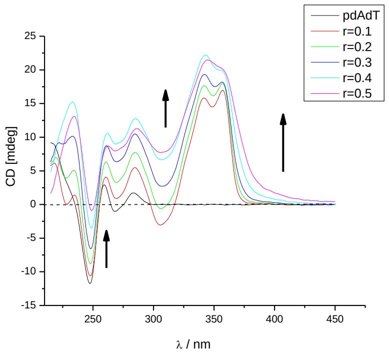
</td><td>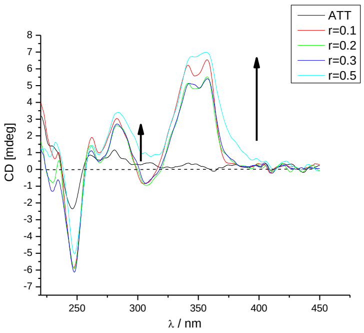
</td></tr>
<tr><td>5</td><td>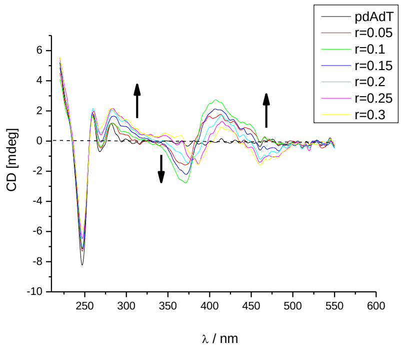
</td><td>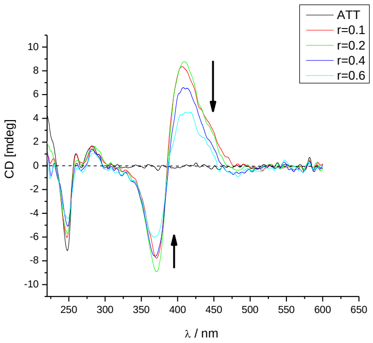
</td></tr>
<tr><td>6</td><td>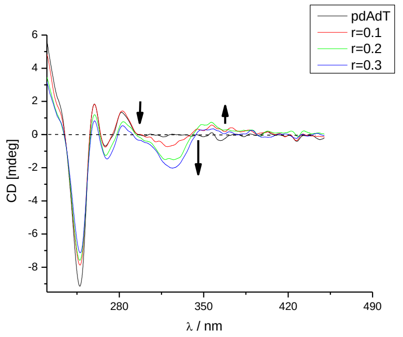
</td><td>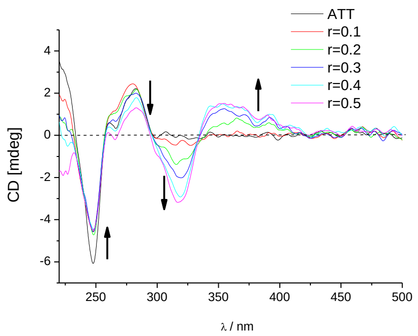
</td></tr>
</table>

Figure S34. CD titrations of poly dA – poly dT ($c = 3.0 \times 10^{-5}$ mol dm^{-3}) and ATT triplex ($c = 3.0 \times 10^{-5}$ mol dm^{-3}) with **1**, **3**, **5** and **6** at molar ratios $\boldsymbol{r}$ = [compound] / [polynucleotide] (pH = 7.0, buffer sodium cacodylate, $I = 0.05$ mol dm^{-3}).

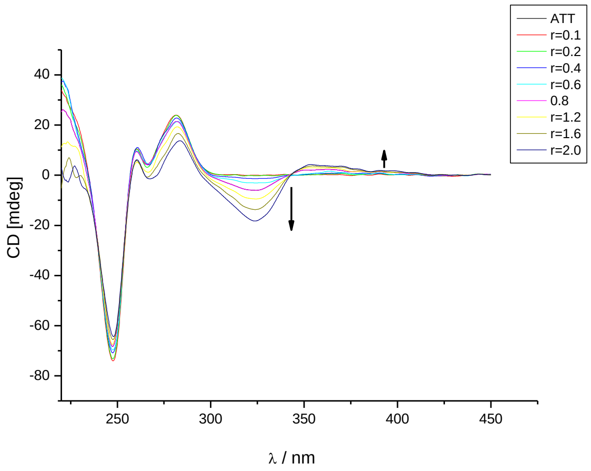

Figure S35. CD titration of ATT 26mer triplex (c = 3.0 × 10^{-5} mol dm^{-3}) and with **6** at molar ratios $\boldsymbol{r}$ = [compound] / [polynucleotide] (pH = 7.0, buffer sodium cacodylate, I = 0.05 mol dm^{-3}).

3. Characterization of 1-9 compounds (NMR, elemental analysis, ESI)

Compound **1**. C. ^{1}H NMR (300 MHz, DMSO-d_6) (δ ppm): 10.70 (br s, 4H, H-Amd), 9.54 (s, 2H,H-Py), 9.13 (s, 1H, H-Py), 8.91 (s, 2H, H-Bt), 8.44 (d, 2H, J = 8.3 Hz, H-Bt), 8.15 (d, 2H, J =8.4 Hz, H-Bt), 4.04 (s, 8H, H-CH2). ^{13}C NMR (75 MHz, D_2O, 70°C) (δ ppm): 167.9, 164.3, 155.7, 149.5, 135.0, 131.8, 127.8, 126.0, 123.7, 123.0, 118.7, 44.7. LC-MS (ESI) m/z: 482.4 [(M + H^+) calcd for free base $C_{25}H_{19}N_7S_2$, 481.11]. Analysis calcd for $C_{25}H_{21}C_{12}N_7S_2$ x $3H_2O$ (608.56): C, 49.34; H, 4.47; N, 16.11, Cl, 11.65. Found C, 49.51; H, 4.54; N, 16.01; Cl, 11.83%.

Compound **2**. ^{1}H NMR (300 MHz, DMSO-d_6) (δ ppm): 10.89 (br s, 4H, H-Amd), 9.02 (d, 1H, J = 4.3 Hz, H-Py), 8.96 (s, 1H, H-Bt), 8.87 (s, 1H, H-Bt), 8.39 (d, 1H, J = 7.2 Hz, H-Py), 8.33 (d, 1H, J = 8.4 Hz, H-Bt), 8.18 (d, 1H, J = 8.6 Hz, H-Bt), 8.00 (d, 1H, J = 8.2 Hz, H-Bt), 7.88 (dd, 1H, J = 4.6 Hz, J = 7.5 Hz, H-Py), 7.65 (d, 1H, J = 8.2 Hz, H-Bt), 4.07 (s, 4H, H-CH2), 4.02 (s, 4H, H-CH2). LC-MS (ESI) m/z: 482.4 [(M + H^+) calcd for free base $C_{25}H_{19}N_7S_2$, 481.11]. Analysis calcd for $C_{25}H_{21}C_{12}N_7S_2$ x $3H_2O$ (608.56): C, 49.34; H, 4.47; N, 16.11, Cl, 11.65. Found C, 49.66; H, 4.35; N, 16.27; Cl, 11.53%.

Compound **3**. ^{1}H NMR (300 MHz, DMSO-d_6) (δ ppm): 8.89 (d, 2H, J = 1.3 Hz, H-Bt), 8.57 (d, 2H, J = 7.8 Hz, H-Py), 8.39 (d, 2H, J = 8.6 Hz, H-Bt), 8.36 (d, 1H, J = 7.8 Hz, H-Py), 8.11 (dd, 2H, J = 1.6 Hz, J = 8.6 Hz, H-Bt), 4.04 (s, 8H, H-CH2). ^{13}C NMR (75 MHz, D_2O/ DMSO-d_6, 80°C) (δ ppm): 170.9, 162.2, 154.3, 146.3, 137.9, 134.1, 123.7, 122.0, 121.3, 121.1, 116.2, 43.0. LC-MS (ESI) m/z: 482.4 [(M + H^+) calcd for free base

$C_{25}H_{19}N_7S_2$, 481.11]. Analysis calcd for $C_{25}H_{21}C_{12}N_7S_2$ x $2H_2O$ (590.55): C, 50.85; H, 4.27; N, 16.60, Cl, 12.01. Found C, 51.12; H, 4.23; N, 16.81; Cl, 11.92%.

Compound **4**. ^{1}H NMR (300 MHz, DMSO-d_6) (δ ppm): 10.57 (s, 2H, -C(NH-$)_2^+$), 8.74 (d, 1H, J = 1.6 Hz, Ar-H), 8.27 (d, 1H, J = 8.6 Hz, Ar-H), 8.11-8.00 (m, 2H, Thioph.-H), 8.02 (dd, 1H, J = 1.7 Hz, J =8.6 Hz, Ar-H), 4.06 (s, 4H, -CH_2CH_2-), 2.31 (s, 3H, CH_3SO_3). ^{13}C NMR (150 MHz, DMSO-d_6) (δ ppm): 164.7 (s), 164.1 (s), 156.2 (s), 136.8 (s), 135.1 (s), 132.7 (d), 129.7 (d), 126.6 (d), 123.7 (d), 123.1 (d), 119.0 (s), 110.5 (s), 44.6 (t, 2C), 39.7 (q). LC-MS (ESI) m/z: 364.5 [(M+H^+) calcd for free base $C_{14}H_{10}BrN_3S_2$, 362.95]. Analysis calcd for $C_{15}H_{14}BrN_3O_3S_3$ (460.39): C, 39.13; H, 3.07; N, 9.13. Found C, 38.98; H, 3.01; N, 9.08 %.

Compound **5**. ^{1}H NMR (300 MHz, DMSO-d_6) (δ ppm): 10.50 (s, 2H, -C(NH-$)_2^+$), 8.69 (d, 1H, J = 1.4 Hz, Ar-H), 8.20 (d, 1H, J = 8.6 Hz, Ar-H), 8.01 (dd, 1H, J = 1.7 Hz, J = 8.6 Hz, Ar-H), 7.94 (d, 1H, J = 4.0Hz, Thioph.-H), 7.66 (d, 1H, J = 5.0 Hz, Thioph.-H), 7.54 (d, 1H, J = 3.4 Hz, Thioph.-H), 7.47 (d, 1H, J = 4.0 Hz, Thioph.-H), 7.17 (dd, 1H, J = 3.7 Hz, J = 4.9 Hz, Thioph.-H), 4.01 (s, 4H, -CH_2CH_2-), 2.33 (s, 3H, CH_3SO_3). ^{13}C NMR (150 MHz, DMSO-d_6) (δ ppm): 165.1 (s), 164.8 (s), 156.8 (s), 142.3 (s), 135.3 (s), 135.0 (s), 133.8 (s), 132.0 (d), 128.6 (d), 127.3 (d), 126.7 (d), 126.0 (d), 125.3 (d), 123.4 (d), 122.7 (d), 118.8 (s), 44.7 (t, 2C), 39.7

(q). LC-MS (ESI) m/z: 368.5 [(M+H$^+$) calcd for free base $C_{18}H_{13}N_3S_3$, 367.03]. Analysis calcd for $C_{19}H_{17}N_3O_3S_4$ (463.62): C, 49.22; H, 3.70; N, 9.06. Found C, 49.10; H, 3.75; N, 9.22 %.

Compound **6**. ^{1}H NMR (300 MHz, DMSO-d$_6$, 50°C) (δ ppm): 10.57 (s, 2H, -C(NH-)$_2$$^+$), 8.81 (s, 1H, Ar-H), 8.37 (d, 1H, J = 8.6 Hz, Ar-H), 8.16 (d, 1H, J = 8.2 Hz, Ar-H), 8.07 (d, 1H, J = 8.8 Hz, Ar-H), 8.00 (d,

1H, J = 8.2 Hz, Ar-H), 7.69-7.61 (m, 2H, Ar-H), 4.08 (s, 4H, -CH_2CH_2-), 2.33 (s, 3H, CH_3SO_3). ^{13}C NMR (75 MHz, DMSO-d$_6$,50°C) (δ ppm): 165.5, 162.7, 155.2, 138.2, 136.9, 135.7, 130.9,129.0, 127.3, 126.8, 124.2, 123.9, 123.8, 123.2, 122.9, 119.9, 45.2 (2C). LC-MS (ESI) m/z: 370.5 [(M+H$^+$) calcd for free base

$C_{18}H_{12}ClN_3S_2$, 369.02]. Analysis calcd for $C_{19}H_{16}ClN_3O_3S_3$ (466.00): C, 46.97; H, 3.46; N, 9.02. Found C, 47.01; H, 3.33; N, 9.27 %.

Compound **7**. ^{1}H NMR (300 MHz, DMSO-d$_6$) (δ ppm): 10.57 (s, 2H, –C(NH–)$_2$$^+$), 8.80 (d, 2H, J =2.0Hz, Ar-H), 8.34 (d, 1H,J =8.6Hz, Ar-H), 8.26 (dd, 1H, J =8.6, 1.8Hz, Ar-H), 8.20 (m, 1H, Ar-H), 8.14 (d, 1H, J =8.7Hz, Ar-H), 8.09–8.01 (m, 2H, Ar-H), 7.70–7.64 (m, 2H, Ar-H), 4.07 (s, 4H, –CH_2–), 2.33 (s, 3H, $CH_3SO_3^-$). ^{13}C NMR (150MHz, DMSO-d$_6$) (δ ppm): 172.2 (s), 164.7 (s), 157.0 (s), 135.1 (s), 134.6 (s),

132.7 (s), 129.5 (s), 129.2 (d), 129.1 (d), 128.4 (d), 128.3 (d),127.9 (d), 127.4 (d), 126.5 (d), 123.9 (d), 123.7 (d), 123.4 (d),118.9 (s), 44.6 (t, 2C). LC–MS (ESI) m/z: 330.1[(M + H$^+$) calcd for free base $C_{14}H_{11}N_3OS$, 329.10]. Analysis calcd for $C_{21}H_{19}N_3O_3S_2$ (425.52): C, 59.27; H, 4.50; N, 9.87. Found C, 59.31; H, 4.55; N, 10.01%.

Compound **8**. ^{1}H NMR (600MHz, DMSO-d$_6$) (δ ppm): 10.61 (s, 2H, –C(NH–)$_2$$^+$), 8.82 (d,1H, J =1.4Hz, Ar-H), 8.35 (d, 1H, J =8.6Hz, Ar-H), 8.06 (dd, 1H, J =1.6Hz, J =8.6Hz, Ar-H), 7.98 (s, 1H, Ar-H), 7.85 (d, 1H, J =7.7Hz, Ar-H), 7.79 (d, 1H, J =8.3Hz, Ar-H), 7.54 (m, 1H, Ar-H), 7.41 (t, 1H, J =7.6Hz, Ar-H), 4.07 (s, 4H, –CH_2–), 2.33 (s, 3H, $CH_3SO_3^-$). ^{13}CNMR (75MHz, DMSO-d$_6$, 70◦C) (δ ppm): 165.5 (s), 161.8 (s), 157.3 (s), 155.8 (s), 149.1 (s), 135.3 (s), 128.2 (s), 127.9 (d), 127.2 (d), 124.7 (d), 124.4 (d), 124.0 (d), 123.3 (d), 119.7 (s), 112.2 (d), 110.0 (d), 45.2 (t, 2C), 40.3 (q). LC–MS (ESI) m/z: 320.1 [(M + H$^+$) calcd for free base $C_{18}H_{13}N_3OS$, 319.08]. Analysis calcd for $C_{19}H_{17}N_3O_4S_2$ (415.49): C, 54.92; H, 4.12; N, 10.11. Found

C, 54.98; H, 4.28; N, 10.07%.

Compound **9**. ^{1}H NMR (300MHz, DMSO-d$_6$) (δ ppm): 11.57 (s, 1H, Ind-H), 10.54 (s, 2H, –C(NH–)$_2$$^+$), 8.70 (s,1H, Ar-H), 8.42 (s, 1H, Ar-H), 8.01 (dd, 1H, J =1.2Hz, J =8.5Hz, Ar-H), 7.93 (dd, 1H, J =1.2Hz, J =8.5Hz, Ar-H), 7.59 (d, 1H, J =8.5Hz, Ar-H), 7.52 (m, 1H, Ar-H), 6.65 (s, 1H, Ar-H), 4.06 (s, 4H, –CH_2–), 2.32 (s, 3H, $CH_3SO_3^-$). ^{13}C NMR (150MHz, DMSO-d$_6$,) (δ ppm): 174.0 (s), 164.7 (s), 157.3 (s), 138.2 (s), 134.7 (s), 128.0 (s),127.6 (d), 126.3 (d), 123.4 (s), 123.2 (d), 122.6 (d), 122.8 (d), 122.6 (d), 117.9 (s), 112.4 (d), 102.6 (d), 44.5 (t, 2C), 39.7 (q). LC–MS (ESI)m/z: 319.1 [(M + H$^+$) calcd for free base $C_{18}H_{14}N_4S$, 318.09]. Analysis calcd for $C_{19}H_{18}N_4O_3S_2$ (414.50): C, 55.05; H, 4.38; N, 13.52. Found C, 55.11; H, 4.51; N, 13.44%.
